# Cutaneous TRPM8‐expressing sensory afferents are a small population of neurons with unique firing properties

**DOI:** 10.14814/phy2.13234

**Published:** 2017-04-05

**Authors:** Michael P. Jankowski, Kristofer K. Rau, H. Richard Koerber

**Affiliations:** ^1^ Department of NeurobiologyUniversity of PittsburghSchool of MedicinePittsburghPennsylvania

**Keywords:** Cold sensation, nociception, sensory neurons, somatosensation

## Abstract

It has been well documented that the transient receptor potential melastatin 8 (TRPM8) receptor is involved in environmental cold detection. The role that this receptor plays in nociception however, has been somewhat controversial since conflicting reports have shown different neurochemical identities and responsiveness of TRPM8 neurons. In order to functionally characterize cutaneous TRMP8 fibers, we used two ex vivo somatosensory recording preparations to functionally characterize TRPM8 neurons that innervate the hairy skin in mice genetically engineered to express GFP from the TRPM8 locus. We found several types of cold‐sensitive neurons that innervate the hairy skin of the mouse but the TRPM8‐expressing neurons were found to be of two specific populations that responded with rapid firing to cool temperatures. The first group was mechanically insensitive but the other did respond to high threshold mechanical deformation of the skin. None of these fibers were found to contain calcitonin gene‐related peptide, transient receptor potential vanilloid type 1 or bind isolectin B4. These results taken together with other reports suggest that TRPM8 containing sensory neurons are environmental cooling detectors that may be nociceptive or non‐nociceptive depending on the sensitivity of individual fibers to different combinations of stimulus modalities.

## Introduction

Neurons in the dorsal root ganglia (DRG) and trigeminal ganglia (TG) sense a variety of thermal, mechanical and chemical stimuli and both A‐ and C‐fibers can respond to these stimuli in varying combinations (Todd and Koerber 2005; Jankowski and Koerber 2010). For example, the most prevalent type of cutaneous C‐fiber found in mice are those that respond to both thermal and mechanical stimulations (Jankowski et al. 2012) (Lawson et al. 2008; Jankowski et al. 2009, 2010). The least prevalent C‐fiber found in these studies was those that responded to a single thermal modality (hot or cold). Several studies have suggested that the thermo‐TRP channels play a role in heat and cold detection (e.g., Dhaka et al. 2007; Lumpkin and Caterina 2007). Transient receptor potential melastatin 8 receptor (TRPM8) specifically has been shown by many groups to be involved in environmental cold detection (McKemy et al. 2002; Bautista et al. 2007; Dhaka et al. 2007), and TRPM8 knockout mice were found to have deficits in cooling detection (McKemy et al. 2002; Bautista et al. 2007). Additionally, this receptor has also been found to play a role in responses to chemical compounds like menthol and icilin (Andersson et al. 2004; Bandell et al. 2006; Brignell et al. 2008; Ding et al. 2008).

Generation of transgenic mice expressing green fluorescent protein (GFP) in TRPM8 neurons (McKemy et al. 2002; Dhaka et al. 2007, 2008) has provided some evidence as to the neurochemical phenotype and function of TRPM8 neurons. In the transgenic line used here, TRPM8 was not found to overlap with any markers of small diameter nociceptors like CGRP, substance P, or IB4. TRPM8‐expressing neurons were also found to innervate the skin at low density and have somas that are among the smallest in the DRG (Dhaka et al. 2008). There was some evidence to suggest that TRPM8 may overlap with the heat sensing ion channel TRPV1 (Dhaka et al. 2008); however, this only appeared to occur after peripheral inflammation. These results are somewhat controversial since other transgenic mice that express GFP in TRPM8 positive neurons display different neurochemistry. TRPM8 was found to overlap with NF200, peripherin and with CGRP in the DRGs, dorsal horn and skin of these other transgenic mice (McKemy et al. 2002). TRPM8 was not found to overlap with IB4 in these animals, but TRPM8‐expressing neurons did respond to menthol in a similar fashion to what was found by Dhaka and colleagues (Dhaka et al. 2007, 2008). Nevertheless, these data do suggest that TRPM8 is expressed in a subset of sensory neurons that respond to cooling and menthol (McKemy et al. 2002; Peier et al. 2002; Bautista et al. 2007; Dhaka et al. 2008; McKemy 2013). However, there has not been a systematic characterization of these neurons' response properties at the single afferent level.

In the following study, we used two ex vivo somatosensory recording preparations to functionally characterize cutaneous TRPM8 neurons in mice genetically engineered to express GFP in TRPM8 DRG cells. Based on previous evidence, we hypothesized that TRPM8 positive neurons projecting to the hairy skin of mice are a unique population of C‐fibers that respond to cooling.

## Methods

### Animals

TRPM8‐eGFP transgenic mice were generated as described previously (Dhaka et al. 2007). Adult (4–6 weeks) male and female mice were used in all experiments. For some experiments, mice from various genetic backgrounds (C57Bl/6, C3HBl/6, Swiss Webster) were used in order to obtain ample cell numbers and better classify the afferent types innervating the hairy skin. Mice were allowed food and water ad libitum and were housed under a 12 h light/dark cycle in a temperature controlled environment. All procedures used in these experiments were reviewed and approved by the Institutional Animal Care and Use Committee at The Scripps Institute and the University of Pittsburgh. Animals were cared for and used in accordance with guidelines of the *U.S. Public Health Service Policy on Humane Care and Use of Laboratory Animals,* the *NIH Guide for the Care and Use of Laboratory Animals* and following institutional AAALAC approved practices.

### Ex‐vivo preparation

The *ex*‐*vivo* somatosensory system preparation has been described in detail previously (Woodbury et al. 2001). Briefly, mice were anesthetized via injection of ketamine and xylazine (90 and 10 mg/kg, respectively) and perfused transcardially with oxygenated (95% O_2_–5% CO2) artificial CSF (aCSF; in mmol/L: 1.9 KCl, 1.2 KH2PO4, 1.3 MgSO4, 2.4 CaCl2, 26.0 NaHCO3, and 10.0 d‐glucose) containing 253.9 mmol/L sucrose at 12–15°C. The spinal cord and the right hindlimb was excised and placed in a bath of aCSF. Hairy skin of the right hindpaw, saphenous nerve, DRGs and spinal cord were isolated. Following dissection, the preparation was transferred to a separate recording chamber containing chilled oxygenated aCSF in which the sucrose was replaced with 127.0 mmol/L NaCl. The skin was pinned out on a stainless steel grid located at the bath/air interface, such that the dermal surface remained perfused with the aCSF while the epidermis stayed dry. The platform served to provide stability during applied thermal and mechanical stimuli. The bath was then slowly warmed to 31°C before recording.

### Blind recording and stimulation

Sensory neuron somata were impaled with quartz microelectrodes (impedance > 150 MΩ) containing 5% Neurobiotin (Vector Laboratories, Burlingame, CA) in 1 mol/L potassium acetate. Orthograde electrical search stimuli were delivered through a suction electrode on the nerve to locate sensory neuron somata innervating the skin. Peripheral receptive fields (RF) were localized with a blunt glass stylus and von Frey hairs. When cells were driven by the nerve but had no mechanical RF, a thermal search was conducted. This was accomplished by applying hot (~52°C) and/or cold (~ 0°C) physiological saline to the skin.

The response characteristics of DRG cells were then determined by applying digitally controlled mechanical and thermal stimuli. The mechanical stimulator consisted of a tension/length controller (Aurora Scientific) attached to a 1 mm diameter plastic disc. Computer controlled 5 sec square waves of 1, 5, 10, 25, 50, and 100 mN were applied to the cell's RF. After mechanical stimulation, a controlled thermal stimulus was applied using a 3 mm^2^ contact area peltier element (Yale Univ. Machine Shop). The cold stimulus consisted of a variable rate cold ramp beginning at 31°C and reaching approximately 4°C, held for 5 sec and slowly allowed to return to 31°C. The heat stimulus consisted of a 12 sec heat ramp from 31–52°C followed by a 5 sec plateau at 52°C. The stimulus then ramped back down to 31°C in 12 sec. Adequate recovery times (approx. 30–45 sec) were employed between stimulations. We have shown previously that multiple stimulus presentations at these intervals is do not result in sensitization (e.g., Lawson et al. 2008). While recording from myelinated nociceptors in many cases, multiple heat applications were made and in some cases the heat ramp was continued to 54°C and held for 5 sec. In other instances, fibers that were unable to be characterized by computer controlled mechanical or thermal stimulation but were characterized by von Frey and/or saline stimuli were also included for determination of instantaneous frequency but not threshold. All elicited responses were recorded digitally for offline analysis (Spike2 software, Cambridge Electronic Design). After physiological characterization, select cells (one per ganglion) were labeled by iontophoretic injection of Neurobiotin (2–3 cells per DRG). Peripheral conduction velocity was then calculated from spike latency and the distance between stimulating and recording electrodes (measured directly along the nerve).

### Ex vivo imaging and recording

TRPM8‐expressing neurons were also specifically targeted for characterization using ex vivo recording coupled with live fluorescent imaging. TRPM8‐eGFP mice were dissected for ex vivo recording as described. All of the remaining bone was then completely removed from the preparation to permit imaging of the DRGs in the live preparation. GFP‐expressing cells were first visualized in the L2/L3 DRGs using FITC optics. The recording electrode was then lowered toward the cell of interest using differential interference contrast (DIC) optics. Once the electrode was close to the cell of interest, the GFP + cell was impaled as described and determined if driven from the nerve. If the GFP+ cell was not found to be driven by the nerve stimulus, another cell was located and the procedure repeated. Once it was determined that the targeted GFP+ cell was driven by the nerve stimulus, mechanical and thermal response properties were attained using an increasing series of von frey hairs and using hot (50–52°C) and cold (31–22°C) saline of varying temperatures. Cells were then filled with Neurobiotin and grouped and characterized as described.

### Menthol application

In an additional set of experiments using the *ex vivo* preparations, we tested for sensitivity to menthol application. First we characterized the cells response properties to mechanical and thermal stimuli as above. Next we applied 30% menthol to the cells cutaneous receptive field using a Q‐tip. This enabled us to apply the menthol solution to a restricted area of skin. In each case we also applied vehicle (50% ETOH) alone to the receptive field alternating, which was applied first. This concentration of menthol dissolved in alcohol is similar to that used previously for cutaneous stimulation (e.g., Namer et al. 2008; Andersen et al. 2015). The location of the area of skin treated with Menthol was recorded on a photograph of the skin and any subsequent cells with receptive fields overlapping one of these areas were not included in this study.

### Tissue processing and analysis of recorded cells

Once a sensory neuron was characterized and intracellularly filled with Neurobiotin, the DRG containing the injected cell was removed and immersion fixed with 4% paraformaldehyde in 0.1 mol/L phosphate buffer (PB) for 30 min at 4°C. Ganglia were then embedded in 10% gelatin, postfixed in 4% paraformaldehyde, and cryoprotected in 20% sucrose. Frozen sections (60 *μ*m) were collected in PB and reacted with fluorescently‐tagged (TRITC) avidin to label Neurobiotin‐filled cells (Vector Laboratories). Next, each section was processed for GFP fluorescence (1:2000; Aves Labs) and IB4 binding (AlexaFluor 647; Molecular Probes, Eugene, OR) or CGRP (1:2000; Chemicon) immunohistochemistry. After incubation in primary antiserum, tissue was washed and incubated in appropriate fluorescently tagged secondary antibodies (1:200; Jackson Immunoresearch). Distribution of fluorescent staining was determined using Olympus FluoView^™^ 500 laser scanning confocal microscope (Olympus America Inc.). Sequential scanning was performed to prevent bleed‐through of the different fluorophores.

Sections of hairy and glabrous hindpaw skin, paw muscles and spinal cord were also dissected, fixed, and sectioned as described above. Skin sections were blocked and incubated in primary antiserum anti‐GFP (Aves 1;2000) overnight at room temperature. After incubation in primary antiserum, tissue was washed and incubated in Cy3‐conjugated goat anti‐chicken secondary antiserum (1:200; Jackson ImmunoResearch). Tissue was analyzed using confocal microscopy.

### Data analysis

One‐way ANOVA tests and posthoc analysis (Tukey) were used to analyze differences in instantaneous frequency in cold responsive C‐fibers. Non parametric, Kruskal–Wallis tests and posthoc analysis (Dunn's test) were used to analyze data associated with mechanical and thermal thresholds of both A‐ and C‐fibers. This information was sorted by neuronal functional type to examine whether or not certain classes of neurons have coherence with regard to the expression of any of the markers tested. *P*‐values were set at *P* < 0.05.

## Results

### Response properties of A‐ and C‐fiber neurons in TRPM8/eGFP mice

A total of 132 primary cutaneous neurons were intracellularly recorded and physiologically characterized from 16 male and female TRPM8/eGFP mice using both blind and targeted intracellular recordings. An additional 24 cold responsive neurons were recorded from 17 male mice which were wildtype for TRPM8. For comparison with the overall population of cold‐sensitive fibers recorded using the same saphenous nerve and stimulation procedures, an additional 216 cold responsive C‐fibers were compiled from previous *ex vivo* studies to characterize the types of cold‐sensitive fibers innervating mouse hairy skin (Albers et al. 2006; McIlwrath et al. 2007; Lawson et al. 2008; Jankowski et al. 2009; Rau et al. 2009; Koerber et al. 2010). Neurons with a conduction velocity of <1.2 m/sec were classified as C‐fibers (Kress et al. 1992), and all others were classified as A‐fibers. Conduction velocities between 1.2 and 10 m/sec were considered to be in the A*δ* range and those ≥10 m/sec were in classified as conducting in the A*β* range (Koltzenburg et al. 1997; McIlwrath et al. 2007). For the purposes of these experiments, we have focused our recordings and analyses specifically on C‐fibers. The C‐fiber classes were as follows: (1) C‐polymodal (CPM), meaning those that responded to mechanical and heat stimuli (CMH) and sometimes cool/cold stimuli (CMHC); (2) C‐mechano (CM), those that responded only to mechanical stimulation of the skin; (3) C‐mechano cool/cold (CMC), those that responded to mechanical and cooling stimuli (but not heating); (4) C‐heat (CH), those that were mechanically insensitive but heat‐sensitive, and (5) C‐cooling/cold (CC), those that were mechanically insensitive but responded to cooling of the skin. Blind recording experiments revealed that small percentages (2%) of recorded cutaneous sensory neurons were TRPM8 positive. Therefore targeted analysis of TRPM8 containing neurons was vital to proper characterization of their response properties. In addition, since TRPM8‐expressing neurons are localized mainly to the small diameter C‐fiber neurons, the analysis described here will focus on this group of cutaneous sensory neurons and when described, will combine results from both blind and TRPM8 targeting experiments.

### Mechanical and thermal response properties of cold/cooling responsive C‐fibers

**Figure 1:**
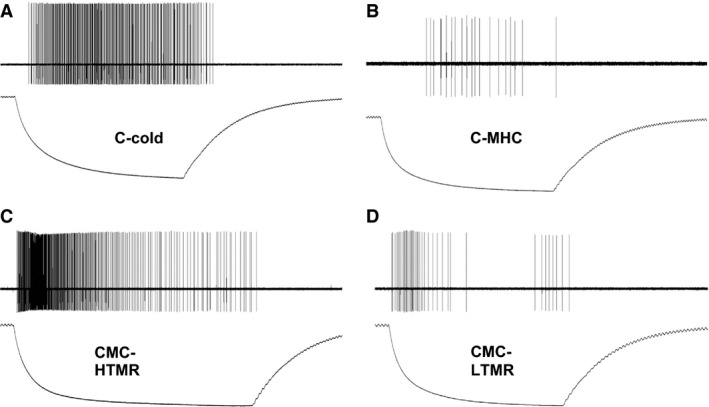
Representative responses of four different cutaneous afferents to cold/ cooling. (A) Mechanically insensitive, cold‐sensitive C‐fibers (C‐cold) respond with vigorous firing to cold/ cooling with sustained responses throughout the duration of stimulation. (B) Polymodal C‐fibers with a cold/ cooling response respond with low frequency firing to cold stimuli. (C) High threshold mechano‐cold C‐fibers (CMC‐HTMR) respond with the highest firing to cooling and reduce responsiveness in the cold range. (D) Low threshold mechano‐cold C‐fibers (CMC‐LTMR) respond to cold/ cooling with low firing frequency.

In previous and current studies using our hairy skin *ex vivo* recording preparations, we found four types of cutaneous C‐fibers that responded to cooling of the skin (Fig. 1). These cold‐sensitive fibers included: (1) those that were mechanically and heat insensitive (CC); (2) those that were also sensitive to mechanical and heat (CMHC; (3) those that were heat insensitive but did respond to high intensity (≥10 mN) mechanical stimulation (CMC‐HTMR); (4) Those fibers that were heat insensitive but did respond to low threshold (≤5 mN) mechanical stimulation (CMC‐LTMR). The most obvious difference between the groups was that a striking difference in the firing rates observed. The CC and CMC‐HTMR groups had instantaneous firing rates that were significantly higher than that seen in CMC‐LTMR and CMCH fibers (Tables 1,2).

**Table 1:** Response properties of functionally characterized cold‐responsive cells from TRPM8‐eGFP mice

	TRPM8‐eGFP mice			
	*n*	GFP+	Cold threshold	Instantaneous frequency
C‐Cold	11	3	20.7 ± 1.61	41.0 ± 13.6^a^
CMC‐HTMR	4	2	20.8 ± 6.7	38.0 ± 18.9^a^
CMC‐LTMR	9	0	20.8 ± 1.52	9.9 ± 3.45
CMHC	19	0	11.0 ± 0.80	2.2 ± 0.57

a *P* < 0.05 versus CMC‐LTMR and CMHC.

**Table 2:** Response properties of functionally characterized cold responsive cells from mice of various genetic backgrounds (All‐TRPM8 + /+)

	All TRPM8 + /+ mice		
	*n*	Cold threshold	Instantaneous frequency
C‐cold	25	16.98 ± 1.34	52.66 ± 7.24^a^
CMC‐HTMR	44	19.03 ± 1.18	62.96 ± 7.97^a^
CMC‐LTMR	44	22.47 ± 0.67	8.63 ± 0.91
CMHC	104	16.04 ± 0.51	4.43 ± 1.18

a *P* < 0.05 versus CMC‐LTMR and CMHC.

### Neurochemical phenotype and response properties of TRPM8‐expressing cells

**Figure 2:**
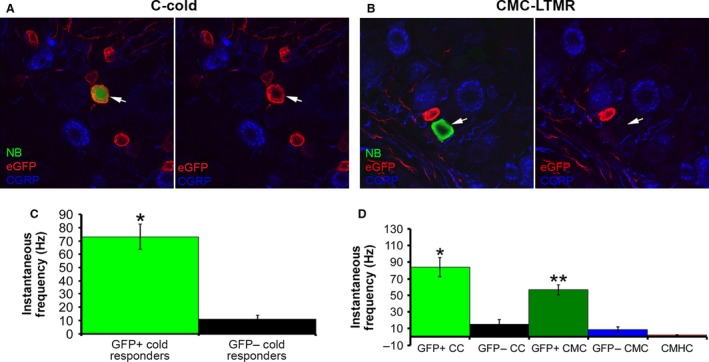
Neurochemical phenotypes of a representative mechanically insensitive, cold‐sensitive C‐fiber (C‐cold) and a low threshold mechano‐receptive, cold‐sensitive C‐fiber (CMC‐LTMR) as identified by ex vivo recording and mean peak instantaneous frequencies to cold/cooling in GFP positive and GFP negative afferents. (A) The C‐Cold neuron filled with neurobiotin (NB) was found to be eGFP positive (red; arrow) and CGRP negative (blue; arrow). (B) The CMC‐LTMR filled with neurobiotin (NB) was found to be both CGRP (blue) and eGFP (red) negative. (C) Mean peak instantaneous frequency to cold/cooling stimuli delivered by the contact pelteir or cold saline was found to be highest in the TRPM8 positive (GFP+) cutanous afferents (73.2 ± 9.5 Hz) compared to other GFP‐ cold responders (11.8 ± 2.8 Hz). * *P* value<0.0000001. (D) Analysis of the different subtypes of cold responders reveals that both GFP+ C‐cold (CC) neurons (84.3 ± 11.5 Hz) and GFP+ C‐mechano cold (CMC) cells (56.6 ± 6.4 Hz) had higher mean peak instantaneous frequencies to cold/ cooling relative to GFP‐ cells of the same type (GFP‐ CC: 15 ± 5.6 Hz; GFP‐CMC: 8.7 ± 3.2 Hz). Polymodal C‐fibers that also had a cooling response (CMHC) were found to have the lowest instantaneous frequency (2.2 ± 0.6 Hz) to cold/ cooling relative to all other cell types. *P < 0.001 relative to GFP‐CC; **P < 0.002 relative to GFP‐CMC. **It is important to note; however, that there were only two GFP + CMCs found during ex vivo recording. There was no difference between GFP + CC and GFP + CMC neurons.

A total of 27 cutaneous C‐fibers were intracellularly labeled, recovered for processing and immunohistochemically characterized in TRPM8‐eGFP mice from the two recording preparations described. We found that TRPM8 containing neurons were of two functional types; CCs (3/6) and CMC‐HTMRs (2/3). No CMC‐LTMR neurons (0/3) contained TRPM8. Although these two functional classifications of C‐fibers sometimes were found to be immunopositive for CGRP (CC: 2/9; CMC‐HTMR: 1/4; CMC‐LTMR: 0/8), none of the TRPM8‐expressing neurons specifically co‐labeled for this marker (Fig. 2A, B). We also found no overlap in CGRP, IB4 or TRPV1 in any CC, CMC‐HTMR or CMC‐LTMRs. In fact, CC, CMC‐HTMR and CMC‐LTMRs were not found to contain TRPV1 or bind IB4 (not shown). CMHC neurons are also not likely to contain TRPM8 since we have shown in numerous studies that these cells are routinely found to bind IB4 (Lawson et al. 2008; Jankowski et al. 2009).

**Figure 3:**
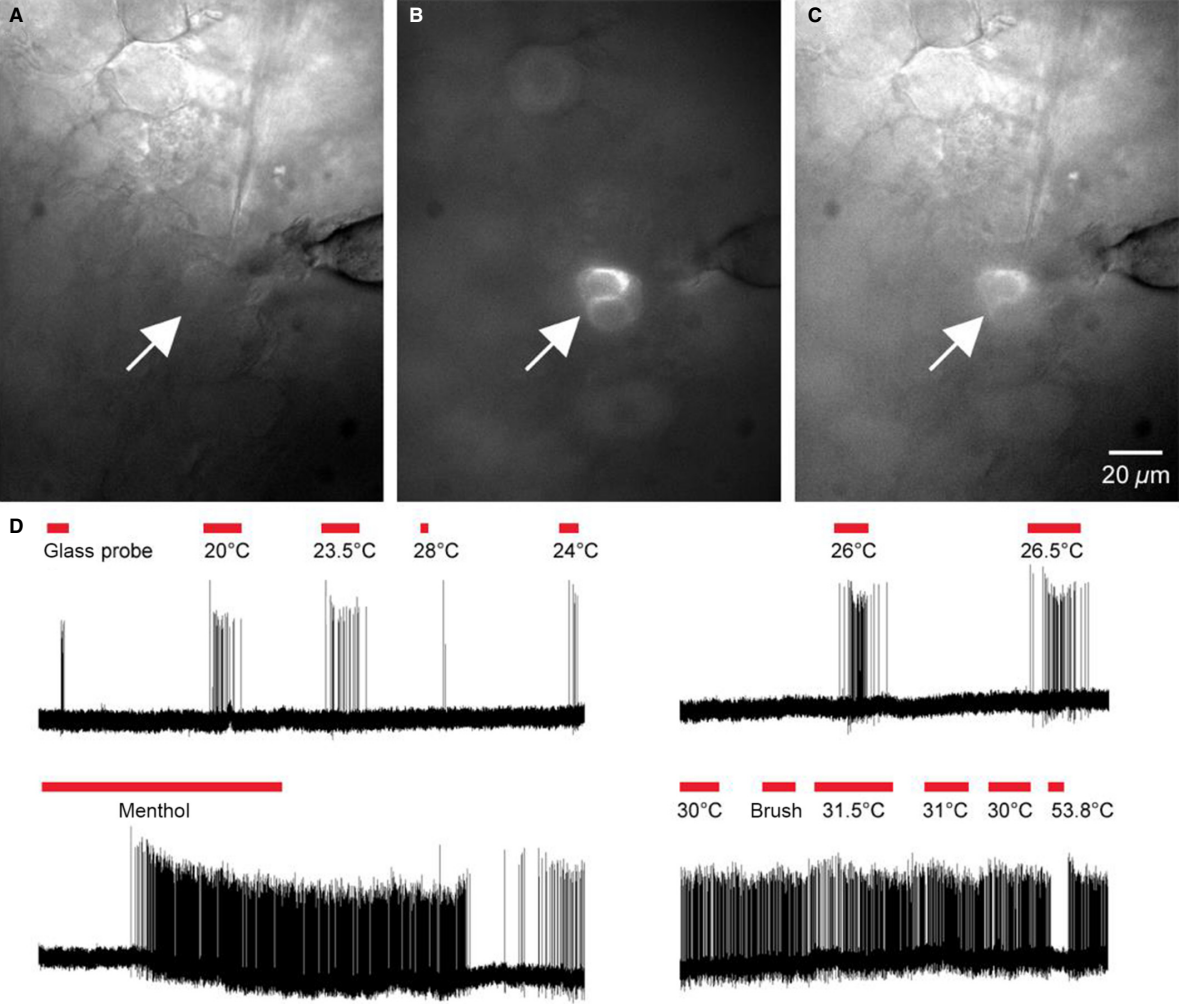
Representative TRPM8 containing cutaneous C‐fiber using targeted ex vivo recording analysis. (A) Bright field image of the L3 DRG during ex vivo recording demonstrates a cell body being penetrated by the recording electrode (shadow). (B) Using FITC optics, the same cell being recorded in panel A was found to be eGFP positive (arrow). (C) Overlay of panels A and B. (D) This cutaneous C‐fiber was found to be sensitive to the glass blunt stylus (glass rod) and a series of cold temperatures ranging from 20°C to 28°C. Application of menthol to the cell's cutaneous receptive field induced rapid firing. The responsiveness to menthol caused the cell to being responding to temperatures above 30°C. Stimulation of the skin with 53.8°C saline ceased firing.

When we assessed TRPM8‐expressing neurons specifically, we found that some responded to mechanical deformation of the skin but always vigorously responded to cold within the cooling temperature range (Figs. 2, 3). Analysis revealed that regardless of whether they were found to be CC or CMC‐HTMRs, TRPM8 +  neurons responded within the cooling range of temperatures with the most vigorous firing to the cold/cooling stimulus (Fig. 2C, D) and had the highest cold/ cooling threshold (24.0 ± 1.83°C). Consistent with previous reports (Dhaka et al. 2008), TRPM8‐expressing cells were also responsive to menthol (Fig. 3D). Interestingly, with the cells we were able to further characterize, application of menthol to the cell's receptive field caused the temperature threshold of activation to increase to temperatures that were apparently above the bath temperature as the cells exhibited continuous high frequency firing which was only briefly interrupted by application of hot saline to the cells receptive field (Fig. 3D). Overall, CC (20.7 ± 1.61°C), CMC‐HTMR (20.8 ± 6.7°C) and CMC‐LTMR (20.8 ± 1.52°C) neurons from the TRPM8‐eGFP mice had average cold thresholds in the cooling range while CMHC neurons began firing to colder temperatures (11.0 ± 0.8°C) similar to what we found in wild type mice.

### Response to menthol

To further examine this population and the relationship between menthol sensitivity and firing frequencies to cold stimulation, we recorded from an additional 24 cold‐sensitive cutaneous C‐fibers in 17 mice that were WT for TRPM8 (TRPM8 + /+). Once the cold response was recorded a Q‐tip soaked in 50% ethanol was applied to the receptive field. The receptive field was rinsed with saline then 30% menthol (dissolved in 50% ethanol) was applied to the same location. None of the cells responded to application of ethanol, however, five out of the 24 cells responded to menthol application. This response was typically the same as that depicted in Figure 3, however the frequency and duration of the response varied. Of these five cells three were classified as CC and two as CMC fibers. Of the 19 non‐responders, seven were characterized as CLTMRs, 8 as CMHCs and 4 were CMCs. The response to cold stimulation was also determined for these fibers prior to chemical applications. While the average cold threshold between the groups did not differ (20.5 ± 3.5°C for menthol‐sensitive vs. 18.6 ± 4.3°C menthol insensitive) the peak instantaneous frequency in response to cold stimulation was significantly higher (*t*‐test, *P* < .01) for the menthols sensitive cells (71.4 ± 37.8 Hz, *n* = 5) compared to the insensitive cells (7.3 ± 3.5 Hz, *n* = 18). Interestingly these results are very similar to that seen for TRPM8/eGFP positive cells in Fig. 2C.

### Localization of TRPM8 axons in the periphery

The cells containing TRPM8 that innervated the distal hairy skin were all of small size and expressed high levels of eGFP. However, there were other cells of larger diameter that were not as intensely labeled with eGFP. We intracellularly recorded from 10 of these cells during the course of these experiments and none were found to be projecting in the saphenous nerve.

**Figure 4:**
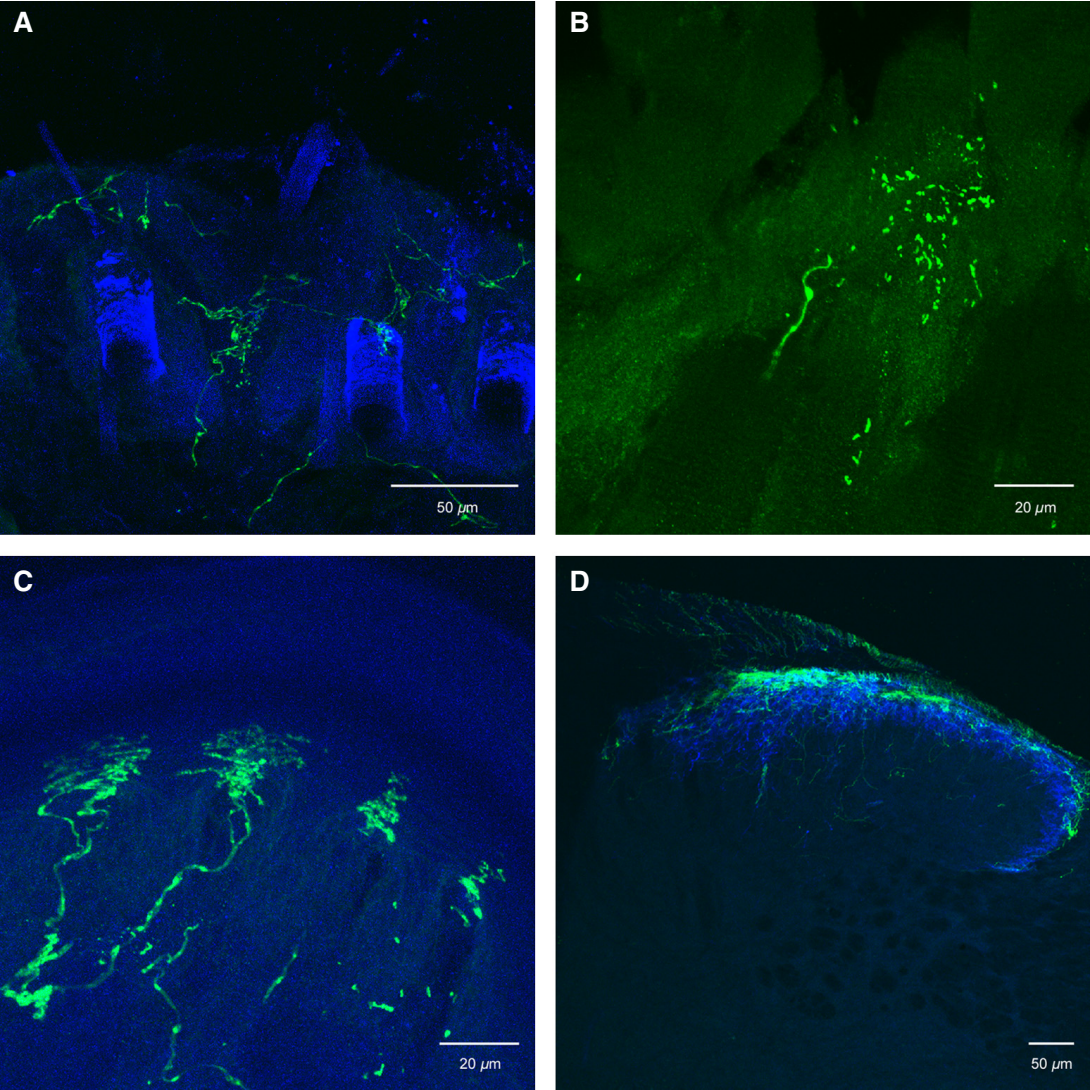
Immunocytochemical analysis of the peripheral and central projections of TRPM8 containing C‐fibers. (A) TRPM8 containing axons (green) project in to the hairy skin dermis and epidermis between hair follicles. (B) These fibers also appear to have punctuate innervation of skeletal (gastrocnemius) muscle. (C) The glabrous skin is also innervated by TRPM8 containing axons whereby the endings have banded patterns in between footpads. (D) TRPM8 axons innervate lamina I of the mouse spinal cord.

In order to assess the peripheral and central innervation patterns of TRPM8 containing sensory neurons, we sampled areas of hairy skin, glabrous skin, interosseous muscles in the paw and spinal cord. As previously reported (Dhaka et al. 2007) we found that these fibers innervated both hairy (Fig. 4A) and glabrous (Fig. 4C) skin and had spinal projections primarily located in Laminae I and the outer part of laminae II (Fig. 4D). In addition, here we found that in the toe and foot pads of the glabrous skin, these fibers had evenly spaced and very structured endings located in the epidermis just above the dermal pegs (Fig. 4C). The location of these unique endings in the foot and toe pads suggests that these afferents are well placed to respond to cold surfaces. Surprisingly, TRPM8‐expressing axons also innervated the muscle with sparse, punctuate endings (Fig. 4B). In a previous report, we have shown that a small subset of muscle C‐fibers innervating these same paw muscles can respond to cold stimuli, suggesting that TRPM8 could be playing a similar roll in muscle (Jankowski et al. 2013).

## Discussion

Previous reports have shown that TRPM8‐expressing DRG neurons are responsive to cooling and menthol stimuli in vitro (Dhaka et al. 2008) and are thought to regulate detection of environmental cooling (Peier et al. 2002; Bautista et al. 2007; Dhaka et al. 2008). Here we have confirmed and expanded upon these results using two ex vivo somatosensory recording preparations. Cutaneous afferents expressing TRPM8 were found to respond to both cooling of the skin and to application of menthol. These cells were also found to be specifically localized to cutaneous C‐fibers that were either mechanically insensitive, but cold/cooling‐sensitive *or* those that were responsive to high threshold mechanical stimuli and cooling. Although both CC and CMC‐HTMRs were occasionally TRPM8 negative, the cells that did contain this channel were found to respond with the highest firing rate to the cooling stimulus compared to all other cold responders.

It is possible that we have underestimated the classification of TRPM8 containing cutaneous C‐fibers since only a small percentage (2%) of recorded cells was found to contain this marker. This is due to the fact that not only is TRPM8 expressed in a small percentage of all DRG neurons, but they are also one of the smallest diameter cell types in the DRG (McKemy et al. 2002; Dhaka et al. 2008). Technical issues of blindly recording from these cells would undoubtedly hinder a full analysis of this fiber type using these methods. Therefore in addition to analyzing C‐fiber function in various mouse strains, we also employed a technique that uses live fluorescent imaging of genetically labeled cells in conjunction with ex vivo recording of individual fiber types (Fig. 3). When using this targeted technique, we still found that TRPM8 containing neurons were confined to the CC and CMC‐HTMR classifications.

Our recent reports have suggested that IB4‐binding cutaneous C‐fibers are mostly CPM neurons (Lawson et al. 2008; Jankowski et al. 2009, 2010), and these cells are known to be largely MrgprD‐expressing (Rau et al. 2009). Although we did not routinely intracellulary stain CPM neurons in the current study, based on our previous data and the response properties and neurochemical phenotypes of TRPM8 containing cells, we would conclude that these cells are at the very least, not CPMs, which would support the hypothesis that these cells are not of the non‐peptidergic nociceptive variety (Snider and McMahon 1998; Dhaka et al. 2007, 2008). Our results would also confirm that these cells are different from the recently described low threshold mechanoreceptive cold/cooling units that express tyrosine hydroxylase (TH) and vesicular glutamate transporter 3 (VGLUT3) and project to deeper laminae of the spinal cord (Seal et al. 2009; Li et al. 2011). Our CMC‐LTMRs are likely to be this cell type. What we may postulate from these results is that cutaneous TRPM8‐expressing neurons are non‐noxious cooling units that can be nociceptive depending on their responsiveness to *mechanical* stimuli. However, it is important to note that while these neurons are responsive to innocuous temperatures, their intense firing during rapid decreases in temp could have a significant impact on pain circuitry in the superficial dorsal horn. In addition, this information they provide on the rate of cooling could be very important in signaling potential harm. Regardless of their role in nociception (Proudfoot et al. 2006; Takashima et al. 2007; Dhaka et al. 2008), the firing patterns appear to reflect their regulation of dynamic and not static responses to cooling.

TRPM8 positive cutaneous C‐fibers were not found to contain markers such as TRPV1, IB4 or CGRP, consistent with previous reports (Dhaka et al. 2008) but in contrast to other studies using different transgenic technologies (McKemy et al. 2002). It has been suggested that the lack of expression of these markers in TRPM8 containing neurons would designate this fiber type as non‐nociceptive (Woolf and Ma 2007). In terms of the cooling response, our data would agree with this notion; however, the fact that we see a small percentage of TRPM8 containing cutaneous afferents that responded to a mechanical stimulus that would be considered noxious (CMC‐HTMRs), challenges this hypothesis and also disputes previously held notions that cooling units do not respond to any other noxious or non‐noxious stimulus (Hensel 1981).

It has been reported that the small percentage of TRPM8 containing neurons that also express TRPV1 (McKemy et al. 2002; Dhaka et al. 2008), may increase under certain injury conditions (Peier et al. 2002; Story et al. 2003; Takashima et al. 2007). Our data would suggest that these dually‐labeled cells are not hairy skin afferents as none of our TRPM8‐expressing fibers contained TRPV1. These TRPM8 + / TRPV1 +  afferents may more likely be glabrous or muscle afferents. We have demonstrated here that TRPM8 containing sensory neurons project to both glabrous skin *and* muscle with unique structural endings (Fig. 4), and TRPV1 fibers are known to project to the glabrous skin but have a denser innervation of the muscle (Christianson et al. 2006). No reports to date have performed a systematic analysis of cold/cooling responses in skeletal muscle; however, it has been reported that TRPM8 may mediate vascular tone (Johnson et al. 2009).

In contrast, TRPM8‐expressing neurons densely innervate the tooth pulp and have been hypothesized to potentially play a role in oral cold pain (Takashima et al. 2007). An alternative hypothesis that has been presented previously (Dhaka et al. 2008) suggested two labeled lines of TRPM8‐expressing neurons: one that is non‐noxious and one that is nociceptive based on the co‐labeling with TRPV1. This is supported by earlier studies showing two distinct subsets of TRPM8‐expressing neurons: one that is capsaicin and menthol‐sensitive and one that is insensitive to these chemicals (Xing et al. 2006).

Several other studies have attempted to designate TRPM8 containing neurons as nociceptive or non‐nociceptive with conflicting results. Although TRMP8 has been described as an environmental cooling detector in naïve mice (McKemy et al. 2002; Bautista et al. 2007; Dhaka et al. 2008) it may also be involved in cold nociception since TRPM8 knockout mice display altered preference to temperatures as low as 5°C compared to wild type mice (Knowlton et al. 2010). In addition, the role that this receptor plays in nociception may change under specific types of injuries. For example, chronic constriction injury (CCI) has been shown to increase TRPM8 expression (Frederick et al. 2007) and the number of menthol/cooling/capsaicin responsive fibers (Xing et al. 2007). Conversely, sciatic nerve injury has been shown to decrease the expression of this receptor (Staaf et al. 2009), which may suggest that TRPM8 may only play a role in noxious cold detection under certain injury conditions. Our data may suggest a more tissue‐dependent and stimulus modality line of function. Whether TRPM8 fibers are noxious or non‐noxious may depend on the compilation of stimulus modalities present in these afferents along with the tissue type and density at which they are innervating. The earlier study by Xing and colleagues (Xing et al. 2006) supports the notion that modality may dictate whether TRPM8 cells are nociceptive or non‐nociceptive. A more comprehensive analysis of the role of the two types of TRPM8 cells described here in different tissue specific afferents; however, will be crucial to our full understanding of this receptor.

## Conflict of Interest

None declared.
